# Essential Oils as an Antifungal Alternative to Control Several Species of Fungi Isolated from *Musa paradisiaca*: Part III

**DOI:** 10.3390/microorganisms13071663

**Published:** 2025-07-15

**Authors:** Maritza D. Ruiz Medina, Jenny Ruales

**Affiliations:** 1 Departamento de Ciencias de Alimentos y Biotecnología (DECAB), Escuela Politécnica Nacional (EPN), Quito 170143, Ecuador; jenny.ruales@epn.edu.ec; 2 Carrera de Alimentos, Universidad Politécnica Estatal del Carchi (UPEC), Tulcán 040102, Ecuador

**Keywords:** oregano, rosemary, clove, thyme, basil, cinnamon, antifungal alternative, post-harvest management, banana

## Abstract

Essential oils (EOs) are widely recognized for their antifungal properties, but their efficacy against specific phytopathogenic fungi associated with banana (*Musa paradisiaca*) rot remains underexplored. This study aimed to evaluate the antifungal potential of EOs from *Origanum vulgare*, *Salvia rosmarinus*, *Syzygium aromaticum*, *Thymus vulgaris*, *Cinnamomum verum*, and *Ocimum basilicum* against five fungal species isolated from infected banana peels. Fungal isolates were obtained using PDA medium supplemented with chloramphenicol and were purified by weekly subculturing. Morphological and microscopic characterization was complemented by molecular identification based on ITS sequencing and phylogenetic reconstruction using Neighbor-Joining and UPGMA methods in MEGA v11. In vitro and ex vivo antifungal assays were performed at EO concentrations ranging from 200 to 1000 ppm. Thyme oil exhibited the strongest inhibitory effect, with complete growth suppression at 1000 ppm. Cinnamon and oregano also demonstrated effective inhibition at 600 ppm, while clove, rosemary, and basil were markedly less effective. Statistical analysis confirmed significant effects of EO type and concentration on fungal growth (*p* < 0.001). Molecular results showed strong phylogenetic support for isolate identification, with bootstrap values above 93% in most clades. These findings support the selective use of specific EOs as sustainable alternatives to synthetic fungicides in the postharvest management of banana diseases and provide a molecularly supported basis for their targeted application in integrated control strategies.

## 1. Introduction

Bananas (*Musa paradisiaca*) are among the most significant crops globally due to their substantial economic and nutritional value, particularly in tropical and subtropical regions. Ecuador is one of the leading banana exporters, and the production of bananas serves as a cornerstone of both the local and national economy [1,2]. Bananas are known for their high quality, flavor, and nutritional content (including vitamins, minerals, and carbohydrates) [3,4].

Despite their importance, banana production faces significant challenges, particularly in the postharvest stage, due to fungal diseases such as crown rot, vascular wilt, and fruit decay [5]. These fungal infections compromise both the quality and shelf life of the fruit [6,7,8]. These diseases are responsible for considerable economic losses each year and hinder global banana trade, especially in regions with high humidity and warm temperatures that favor fungal proliferation [9,10]. Among the fungi are *Fusarium pseudocircinatum*, *Fusarium verticilloides*, *Colletotrichum tengchongense*, *Fusarium napiforme*, and *Verticillium dahliae*, which affect the banana plant’s vascular system, leading to reduced fruit yield and quality [11,12,13].

The control of these fungal pathogens has traditionally relied on synthetic fungicides, but growing concerns about their environmental impact, development of fungicide resistance, and potential health risks have spurred interest in finding alternative, more sustainable methods [14,15]. The EOs, derived from plants such as *Origanum vulgare* (oregano), *Syzygium aromaticum* (clove), *Cinnamomum verum* (cinnamon), *Thymus vulgaris* (thyme), and *Salvia rosmarinus* (rosemary), have emerged as promising natural antifungal agents [16,17,18].

These oils contain bioactive compounds such as carvacrol, eugenol, cinnamaldehyde, and thymol, which have demonstrated potent antimicrobial and antifungal properties in various agricultural contexts [19,20,21]. However, while EOs have been studied for their antimicrobial activity, there remains a significant gap in the literature regarding their effectiveness specifically against the fungus pathogens affecting bananas, particularly under postharvest conditions [1,22,23].

The antifungal effects of these EOs are primarily explained by their ability to disrupt the fungal cell membrane, inhibit ergosterol biosynthesis, and induce oxidative stress. Disruption of the fungal cell membrane increases its permeability, allowing ions, proteins, and essential metabolites to leak out, ultimately resulting in the death of the cell [20,24]. Cinnamaldehyde, carvacrol, and thymol have been shown to disrupt ergosterol biosynthesis, which is essential for maintaining the structure and function of the fungal membrane [25].

Rosmarinic acid in rosemary oil and eugenol in basil oil induce the production of reactive oxygen species (ROS), which causes oxidative damage to cellular components, leading to cell death [14,16]. These mechanisms of action are particularly relevant for the control of *Fusarium*, *Colletotrichum*, and *Verticillium* species, which are responsible for banana postharvest diseases [24,26].

Among the EOs considered in this study, oregano oil (*Origanum vulgare*) stands out due to its high carvacrol content (60–80%), a phenolic compound known for its potent antimicrobial and antioxidant properties [17,27]. Carvacrol has been shown to disrupt fungal cell membranes by integrating into the lipid bilayer, increasing membrane permeability and leading to cell death [28]. Thymol shares similar mechanisms of action; p-cymene and γ-terpinene also contribute synergistically to its antifungal activity [29].

*Syzygium aromaticum* (clove) EOs contain eugenol (70–85%), a compound with antifungal properties. Eugenol acts by denaturing proteins, including enzymes critical to fungal metabolism, and by inhibiting vital cellular processes such as ATP synthesis and cellular respiration, ultimately resulting in fungal cell death [26,30]. The antioxidant activity had IC50 values of 4.82 ± 0.06 × 10^−2^ µg/mL and EC50 values of 3.47 ± 0.2 × 10^−2^ µg/mL. The results indicate that EOs exhibit significant in vitro antioxidant activity and moderate antibacterial activity [31].

Cinnamon EOs (*Cinnamomum verum*), rich in cinnamaldehyde (60–70%) and eugenol (5–15%), are another promising candidate for antifungal control [19,32]. Cinnamaldehyde disrupts fungal cell membranes and inhibits ergosterol biosynthesis, a component of the fungal cell membrane, thereby impairing fungal growth and nutrient transport. It has been shown to be effective against *Fusarium* species wilt in bananas [33,34]. A study examined the antifungal activity of EOs and their compounds on the growth of *Fusarium* spp. isolated from papaya rot [35].

Thyme oil (*Thymus vulgaris*), which contains high concentrations of thymol (30–50%) and carvacrol (10–20%), has demonstrated broad-spectrum antifungal activity by integrating into fungal cell membranes, causing membrane leakage and disrupting the integrity of the cell [36,37]. *Ocimum basilicum*, or basil, is also from the *Lamiaceae* family and is rich with bioactive compounds such as eugenol, rosmarinic acid, and flavonoids, and is primarily valued for its antioxidant properties [38]. Additionally, it offers anticancer, antimicrobial, anti-inflammatory, and antioxidant benefits [39].

Finally, rosemary oil (*Salvia rosmarinus*), with 1,8-cinee (20–50%) and carnosol (5–15%), induces oxidative stress in fungal cells. The generation of ROS leads to oxidative damage to fungal proteins, lipids, and DNA, accelerating fungal cell death [16].

While EOs show great promise as natural antifungal agents, it is crucial to consider their potential phytotoxicity. High concentrations of EOs can cause damage to plant tissues, leading to symptoms such as chlorosis, necrosis, and reduced growth, particularly when applied directly to the plant [14]. Previous studies have highlighted the importance of determining the optimal concentration for antifungal efficacy, ensuring that EOs can be used effectively without harming the plant. Emulsions containing EOs in concentrations ranging from 0.1 to 1% have been reported as safe for use on postharvest fruits. Specific tolerance tests on the fruit of the banana are necessary to ensure the safety of these concentrations. It is essential to balance antifungal efficacy with phytotoxicity to ensure the safe and effective application of EOs [24].

In conclusion, this study evaluates the antifungal potential of EOs applied in the postharvest of banana for protection against *Fusarium pseudocircinatum*, *Colletotrichum tengchongense*, *Suarium napiforme*, *Fusarium verticilloides*, and *Verticillium dahlia*, which affect bananas. This research will contribute valuable insights into the potential use of EOs as natural, sustainable alternatives to synthetic fungicides.

## 2. Materials and Methods

### 2.1. Isolation and Purification of Microorganisms

In this study, *Musa paradisiaca* was used, specifically Ecuadorian bananas of export quality. The fruits were exposed to environmental conditions until visible signs of deterioration developed, including fungal growth on the peel covering at least 50% of the banana’s surface.

The isolation and purification of pathogenic fungi from infected banana peels (*Musa paradisiaca*) were conducted using PDA (Difco™, Detroit, MI, USA) medium, which is widely recognized for its ability to support fungal growth. PDA was prepared by dissolving 39 g of the medium in 1 L of distilled water, followed by sterilization in an autoclave at 121 °C for 15 min [40,41]. To prevent bacterial contamination, chloramphenicol (Merck, Ecuador) was added at a final concentration of 0.5 g/L [42,43].

Samples were collected from 30 bananas showing visible signs of fungal infection on the peel. Approximately 20 g of infected tissue was aseptically removed from the sample. The peel fragments were immersed in sterile distilled water and manually agitated for 2 min. This process was repeated twice with fresh sterile water, discarding the used water after each step [44,45,46].

For spore extraction, the cleaned peel fragments were transferred to 200 mL Erlenmeyer flasks containing a 0.05% (*v*/*v*) aqueous solution of Tween 80 (RMC, Quito, Ecuador). This surfactant facilitated the release of fungal spores from the infected tissue. The suspension was vortexed for 2 min to ensure homogeneity [47,48]. Four serial dilutions were prepared from the initial suspension, using a 0.1% aliquot of the previous dilution at each step to ensure proper spore distribution mixing in a vortex [49].

Aliquots of 0.1 mL from each dilution were plated onto Petri dishes containing PDA supplemented with 0.05% chloramphenicol. The plates were incubated at 25 ± 2 °C with a 12:12 h light/dark cycle at 75 ± 5% relative humidity [50]. Visible fungal colonies were carefully selected with a sterile loop to avoid cross-contamination and were subcultured weekly onto fresh PDA plates until pure cultures were obtained [49,51]. These purified isolates were subsequently used for both macroscopic and microscopic morphological characterization and preserved for molecular identification through ITS sequencing.

### 2.2. Morphological Identification

The purified fungal strains were analyzed macroscopically and microscopically to confirm their identity. Pure cultures were maintained on PDA at 25 °C for short-term studies and preserved at −80 °C in a 15% glycerol solution for future molecular studies.

The morphological identification of fungal pathogens involved a comprehensive analysis of macroscopic and microscopic characteristics to determine their genus [46]. Pure fungal cultures were prepared and evaluated weekly over a four-week period, with each analysis conducted in triplicate to ensure reproducibility. These evaluations were performed following inoculation on PDA medium. The macroscopic examination focused on morphological traits, including colony shape, elevation, edges, color, texture, and surface appearance [52].

Observations of the upper and lower surfaces of the Petri dishes provided additional insights into fungal colony characteristics. The recorded macroscopic features were systematically compared against bibliographic information obtained from specialized literature, such as books and guides on fungal morphology, to identify the genus of the pathogens [7,53].

Microscopic analysis was conducted to observe fungal reproductive structures, including hyphae, conidia, mycelium, and spores. For this, a conventional method using adhesive tape was employed to collect the aerial mycelium, which was then mounted on microscope slides. These slides were examined for triplicate under a compound microscope with 40X and 60X objective lenses. Detailed observations focused on hyphae, spore formation, and other specialized microscopic structures. Results were cross-referenced with descriptive fungal morphology guides to confirm the findings [54].

### 2.3. Molecular Identification Through DNA Sequencing

Fungal species isolated from *Musa paradisiaca* (banana) were identified molecularly using DNA sequencing methods to ensure precise species identification. Genomic DNA was extracted from pure fungal colonies using the Invitrogen commercial kit (Novogene, Sacramento, CA, USA), strictly adhering to the manufacturer’s instructions. To evaluate the quality and quantity of the extracted DNA, spectrophotometry was conducted using a Nanodrop spectrophotometer (Thermo Fisher Scientific, Waltham, MA, USA), alongside 1% agarose gel electrophoresis to verify DNA integrity [55].

The ribosomal DNA fragment corresponding to the Internal Transcribed Spacer (ITS) region was amplified using the universal primers ITS1 (5′-TCCGTAGGTGAACCTGCGG-3′) and ITS4 (5′-TCCTCCGCTTATTGATATGC-3′). The PCR amplification was optimized in a thermocycler, and the resulting amplicons were visualized on a 1.5% agarose gel stained with ethidium bromide under UV light to confirm amplification. Purified amplicons were subsequently sent to Macrogen Inc. (Seoul, Republic of Korea) for sequencing [47].

The obtained sequences were analyzed using BioEdit software, version 7.0 [56], for alignment and further comparison with publicly available databases, such as the NCBI GenBank https://www.ncbi.nlm.nih.gov/genbank/ (accessed on 8 May 2025). The BLASTn algorithm was employed to identify fungal species based on sequence similarity. Identification was confirmed with a threshold of ≥98% similarity to the deposited sequences in the database [42,47].

Phylogenetic analysis was conducted to elucidate the evolutionary relationships among fungal isolates based on internal transcribed spacer (ITS) region sequences. Multiple sequence alignments were performed using the ClustalW algorithm to ensure accurate alignment of conserved and variable regions. Phylogenetic trees were constructed using both the Unweighted Pair Group Method with Arithmetic Mean (UPGMA) and the Neighbor-Joining (NJ) method, implemented in MEGA version 11 (Molecular Evolutionary Genetics Analysis, Pennsylvania State University, USA) [57]. The UPGMA was selected for its capacity to generate rooted trees under a constant-rate molecular clock assumption, while NJ was employed for its efficiency in producing unrooted trees accommodating heterogeneous evolutionary rates.

Pairwise identity matrices and genetic distances were calculated using the Tamura-Nei substitution model, which accounts for differences in nucleotide substitution rates and unequal base frequencies. Clade support was assessed via bootstrap resampling with 1000 replicates, and bootstrap values ≥ 70% were considered statistically significant.

This integrative methodological approach, combining robust sequence alignment, dual phylogenetic tree reconstruction strategies, genetic distance estimation, and statistical validation, is consistent with established practices in molecular mycology. Furthermore, it is supported by previous studies that validate the ITS region as a reliable genetic marker for fungal identification and phylogenetic inference [20,23,38].

### 2.4. Ex Vivo Fungal Activity

The EOs used in this study were obtained from Green Harmony, a company specialized in the production and distribution of pure and natural. Oregano EOs (*Origanum vulgare*) are sourced from Turkey, cinnamon (*Cinnamomum verum*) from Sri Lanka, clove (*Eugenia caryophyllata*) from Indonesia, thyme (*Thymus vulgaris*) from Japan, basil (*Ocimum basilicum*) from India, and rosemary (*Rosmarinus officinalis*) from Spain. The quality of EOs can be influenced by their geographical origin, which affects their chemical profile and properties [58,59].

The EOs, used at varying concentrations (200, 400, 600, 800, and 1000 ppm), were evaluated for their antifungal activity. These oils were standardized using the widely recognized steam distillation method, known for its ability to preserve the volatile compounds present in plants [60,61]. This technique ensures that the oils maintain their quality and the concentration of active compounds without significant alterations to their chemical profile [62,63]. Oregano EOs (*Origanum vulgare*) were derived from dried leaves, cinnamon (*Cinnamomum verum*) from dried bark, and clove (*Syzygium aromaticum*) from dried flower buds; rosemary (*Salvia rosmarinus*), basil (*Ocimum basilicum*), and thyme (*Thymus vulgaris*) were extracted from fresh leaves and flowers.

Oregano EOs are rich in carvacrol (60–80%), a phenolic compound known for its antimicrobial and antioxidant properties. It also contains thymol (5–10%), p-cymene (5–10%), and γ-terpinene (2–5%), which work synergistically to inhibit microbial growth. Rosemary EOs are primarily composed of 1,8-cineole (20–50%), carnosol (5–15%), rosmarinic acid (2–5%), and α-pinene (10–20%), compounds that have antioxidant, antimicrobial, and potential neuroprotective effects.

Clove EOs are characterized by their high concentration of eugenol (70–85%), followed by acetyl eugenol (5–15%) and β-caryophyllene (5–12%), which contribute to their effectiveness against fungi and bacteria. Thyme EOs contain thymol (30–50%) and carvacrol (10–20%), as well as p-cymene (15–25%) and γ-terpinene (10–15%), which are responsible for its anti-fungal activity.

Cinnamon EOs contain cinnamaldehyde (60–70%) and eugenol (5–15%), known for their antimicrobial and antioxidant properties. Lastly, basil EOs are distinguished by their high eugenol content (50–70%), linalool (10–15%), and methyl chavicol (5–15%), compounds with antimicrobial, anti-inflammatory, and antioxidant properties.

The antifungal activity of fungal pathogens isolated from *Musa paradisiaca* (banana) was assessed in an ex vivo analysis under controlled conditions of approximately 13 ± 1 °C and 92 ± 3% relative humidity. The fungi analyzed included *Fusarium pseudocircinatum*, *Fusarium verticilloides*, *Colletotrichum tengchongense*, *Fusarium napiforme*, and *Verticillium dahliae*, all of which were isolated from banana peel samples. These pathogens were selected based on their relevance in postharvest diseases of bananas during storage of 15 fungi in total.

Fungi were stored for 7 to 10 days before analysis, and inoculum concentrations were standardized to 10^6^ conidia/mL of each inoculum to maintain consistent infection levels [33]. Bananas harvested at physiological maturity were selected for uniform size, color, and absence of visible damage. The fruits were disinfected with a 1% sodium hypochlorite solution for 5 min, rinsed with sterile distilled water, and dried at room temperature [64]. Inoculation was performed using the wound method, where 100 µL of the adjusted fungal inoculum was applied. The fruits were maintained at approximately 13 ± 1 °C and 92 ± 3% relative humidity [65].

Observations were recorded at regular intervals to measure lesion diameter and fungal growth diameter using a millimeter ruler, with four replicates for each treatment. Fungal growth diameter was monitored weekly for up to 6 weeks post inoculation, providing a comprehensive timeline of pathogen progression. The inhibition index and severity of infection were calculated in triplicate, based on the diameter of fungal growth; this analysis enabled the classification of pathogens.

### 2.5. In Vitro Antifungal Activity with Essential Oils

The in vitro antifungal activity of EOs derived from six plant species was evaluated under controlled laboratory conditions. The EOs included oregano (*Origanum vulgare*), rosemary (*Salvia rosmarinus*), clove (*Syzygium aromaticum*), thyme (*Thymus vulgaris*), cinnamon (*Cinnamomum verum*), and basil (*Ocimum basilicum*) [21,66]. These oils were extracted using steam distillation, a standard method for isolating bioactive compounds from plants.

Specifically, oregano oil was obtained from dried leaves, cinnamon oil from dried bark, and clove oil from dried flower buds, while rosemary, basil, and thyme oils were extracted from fresh leaves and flowers. The oils were procured from commercial suppliers and diluted in 0.05% Tween 80 solution to form homogeneous emulsions with concentrations of 200, 400, 600, 800, and 1000 ppm [49].

The emulsions were incorporated into cooled PDA medium before solidification. The medium was inoculated with fungal pathogens and incubated at 25 ± 2 °C and 75 ± 5% relative humidity, with a photo period of 12 h of light and 12 h of darkness and monitored daily to observe the inhibitory effects of the EOs (batch 20230516) on fungal growth. Negative controls included PDA medium without EOs, allowing for baseline fungal growth comparisons.

Each experimental condition was conducted in quadruplicate with three independent replicates, and the percentage of growth inhibition was assessed every 24 h. Observations focused on determining the concentration of each EOs that effectively inhibited fungal growth. The study employed a 6 × 5 mixed factor model to analyze the antifungal activity, with six types of EOs and five concentration levels as independent variables, and the percentage of growth inhibition as the dependent variable.

Analyses identified the most effective EOs and concentration for fungal inhibition. Results demonstrated significant variability in the antifungal efficacy of oils, providing valuable insights into their potential applications as natural antifungal agents in biocontrol and agriculture. By evaluating a diverse range of EOs at varying concentrations, this study contributes to the development of eco-friendly alternatives for managing pathogens.

Table 1 presents the experimental conditions used to evaluate the antifungal activity of essential oils against banana fungi. It includes details on the oil concentrations, the fungal species tested, and the evaluation methods, aiming to provide reliable data on the potential of essential oils for controlling fungal diseases in bananas.

## 3. Results

### 3.1. Morphological Identification

**Figure 1:**
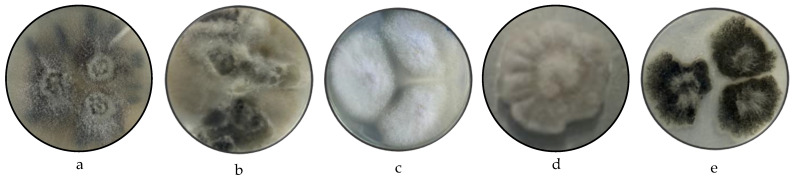

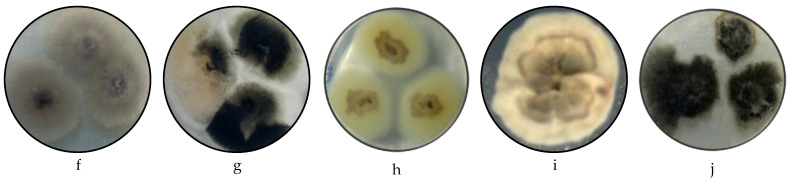
Macroscopy images of (**a**) *Fusarium pseudocircinatum*, (**b**) *Colletotrichum tengchongense*, (**c**) *Fusarium verticilloides*, (**d**) *Fusarium napiforme*, (**e**) *Verticillium dahliae* considering the appearance of the front side and (**f**) *Fusarium pseudocircinatum*, (**g**) *Colletotrichum tengchongense*, (**h**) *Fusarium verticilloides*, (**i**) *Fusarium napiforme*, and (**j**) *Verticillium dahliae* of reverse side.

Figure 1 presents the pure fungi isolated from banana peel rot on a selective medium (PDA + Chloramphenicol), stored in PDA at approximately 25 °C. It also includes macroscopic images of the front and reverse sides of *Fusarium pseudocircinatum*, *Colletotrichum tengchongense*, *Fusarium napiforme*, *Fusarium verticilloides*, and *Verticillium dahliae*.

**Figure 2:**
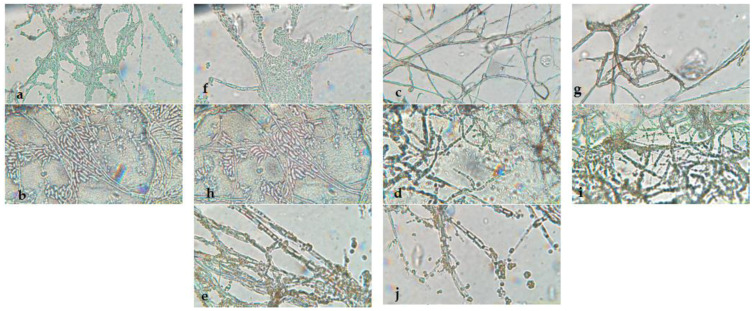
Microscopy images of (**a**) *Fusarium pseudocircinatum*, (**b**) *Colletotrichum tengchongense*, (**c**) *Fusarium verticilloides*, (**d**) *Fusarium napiforme*, (**e**) *Verticillium dahliae* and (**f**) *Fusarium pseudocircinatum*, (**g**) *Colletotrichum tengchongense*, (**h**) *Fusarium verticilloides*, (**i**) *Fusarium napiforme,* and (**j**) *Verticillium dahliae*.

Figure 2 shows the aerial mycelium of the fungi. These observations offer visual details that complement the morphological analysis and microscopic images viewed under 40X and 60X magnifications.

### 3.2. Molecular Identification Through DNA Sequencing

A phylogenetic tree was constructed using ITS region sequences to determine the evolutionary relationships among fungal isolates obtained from *Musa paradisiaca*. The analysis was performed using the Neighbor-Joining method with the Tamura–Nei substitution model in MEGA v11, and clade support was assessed through 1000 bootstrap replicates.

**Figure 3:**
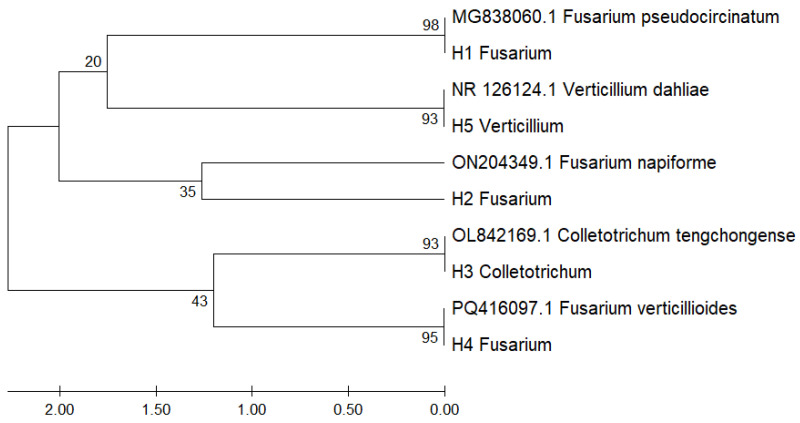
Phylogenetic tree based on ITS region sequences of fungal isolates associated with *Musa paradisiaca*.

The resulting topology, shown in Figure 3, revealed distinct and well-supported groupings that confirm species-level identity. Isolate H1 clustered with *Fusarium pseudocircinatum* (MG838060.1) with a bootstrap value of 98%, indicating high confidence in its molecular classification. Isolate H5 grouped with *Verticillium dahliae* (NR126124.1), supported by 93%, while H2 aligned with *Fusarium napiforme* (ON204349.1), albeit with a moderate bootstrap of 35%. Isolate H3 clustered with *Colletotrichum tengchongense* (OL842169.1) with 93% support, and H4 grouped strongly with *Fusarium verticillioides* (PQ416097.1) with a bootstrap value of 95%.

These phylogenetic relationships support the accuracy of species identification based on ITS sequences and align with established classifications for pathogenic fungi associated with tropical crops. The high bootstrap values (≥93%) observed in four of the five clades reinforce the reliability of the ITS marker in resolving taxonomic placement at the species level.

The molecular identification of fungal isolates was carried out through BLASTn comparison of ITS region sequences against the NCBI GenBank database. The results confirmed high sequence identity values (≥98.7%) between the isolates and their respective reference strains, supporting precise species-level identification.

As detailed in Table 2, the highest similarity was observed between the isolate identified as *Colletotrichum tengchongense* and its reference sequence OL842169.1, with 99.66% identity. Similarly, *Fusarium pseudocircinatum* (MG838060.1) and *Verticillium dahliae* (NR126124.1) showed identity values of 99.54% and 99.38%, respectively. The lowest match observed—though still highly reliable—was with *Fusarium napiforme* (ON204349.1), which aligned with 98.71% identity.

All identifications exceeded the commonly accepted 98% threshold for species-level resolution using ITS sequences, reinforcing the validity of the isolates’ taxonomic assignment.

To evaluate the genetic similarity between fungal isolates and their respective reference strains, a pairwise distance matrix was generated based on ITS sequences. Table 3 presents the calculated genetic distances expressed as percent divergence. The results reveal a strong phylogenetic relationship between each isolate and its corresponding species, indicating a high degree of sequence conservation.

Isolate H1 exhibited a minimal distance (0.002) from *Fusarium pseudocircinatum* (MG838060.1), while H3 differed by only 0.002 from *Colletotrichum tengchongense* (OL842169.1), and H4 by 0.005 from *Fusarium verticillioides* (PQ416097.1). Similarly, H5 showed a very low divergence (0.004) from *Verticillium dahliae* (NR126124.1). These extremely low values (<0.01) confirm a high degree of identity and support the species-level classification of the isolates.

In contrast, the greatest distances were observed between phylogenetically distant species, such as *Fusarium pseudocircinatum* and *Colletotrichum tengchongense*, which showed a divergence of 5.62. These findings are consistent with the topology of the phylogenetic tree (Figure 3) and underscore the value of pairwise distance analysis as a complementary approach to validating evolutionary relationships.

### 3.3. Fungal Activity Ex Vivo

**Figure 4:**
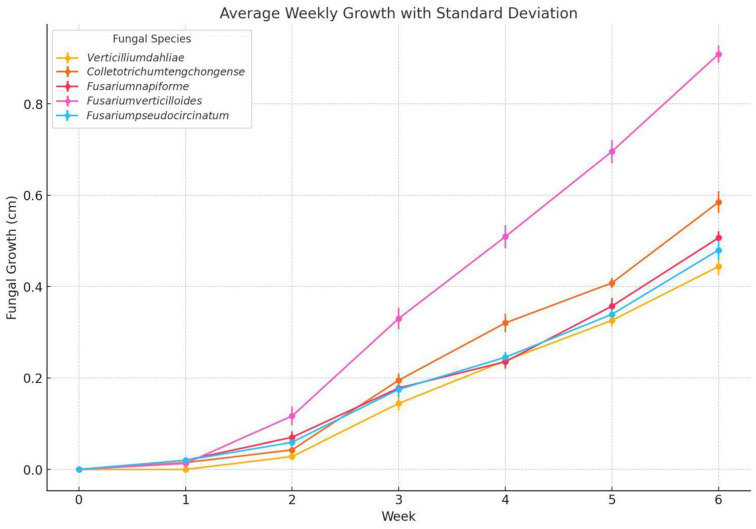
Fungal growth (cm) during 6 weeks in 20 banana samples inoculated with *Fusarium pseudocircinatum*, *Colletotrichum tengchongense*, *Fusarium verticilloides*, *Fusarium napiforme*, and *Verticillium dahlia*, stored at 13 °C and 95% HR approximately.

The severity of fungal infection in *Musa paradisiaca* samples was assessed through ex vivo analysis, in which 20 banana fruits were monitored over a six-week period. Fungal development was evaluated to be of five species: *Fusarium pseudocircinatum*, *Colletotrichum tengchongense*, *Fusarium napiforme*, *Fusarium verticillioides*, and *Verticillium dahliae*. Figure 4 provides a visual representation of radial growth progression over time.

Statistical analysis was conducted using an ANOVA framework to evaluate the effects of treatment, concentration, and time on fungal development. Statistically significant differences were observed between treatments (*p* < 0.05), indicating that both the type and concentration of treatment had a notable influence on growth inhibition. Furthermore, a two-way ANOVA (species and time) revealed that fungal growth was significantly affected by species (F = 219.96, *p* < 0.001), week (F = 2034.58, *p* < 0.001), and their interaction (F = 24.94, *p* < 0.001), indicating distinct growth trajectories among taxa.

A one-way ANOVA followed by Tukey’s HSD post hoc test performed at week six showed that *F. verticillioides* exhibited significantly greater radial growth compared to all other species (*p* < 0.001), supporting its potential as a dominant colonizer under in vitro conditions. In contrast, no statistically significant differences were found among *F. pseudocircinatum*, *V. dahliae*, and *F. napiforme*, while *C. tengchongense* displayed an intermediate growth pattern. These findings underscore the importance of multispecies monitoring and robust statistical validation in postharvest pathogenicity studies.

### 3.4. In Vitro Antifungal Activity with Essential Oils

**Figure 5:**
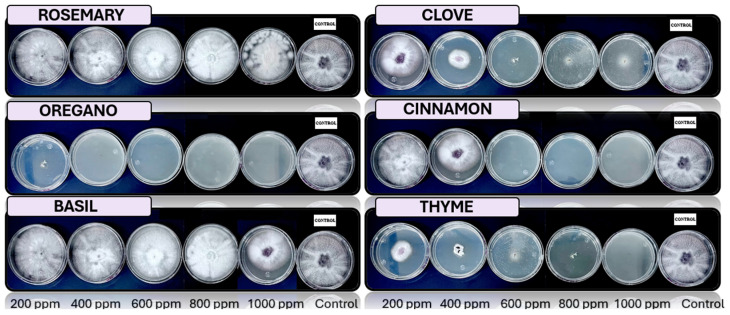
In vitro analysis of *Fusarium pseudocircinatum* in PDA medium supplied with basil, cinnamon, clove, oregano, rosemary, and thyme essential oils at various concentrations of 200, 400, 600, 800, and 1000 ppm (*n* = 12).

**Figure 6:**
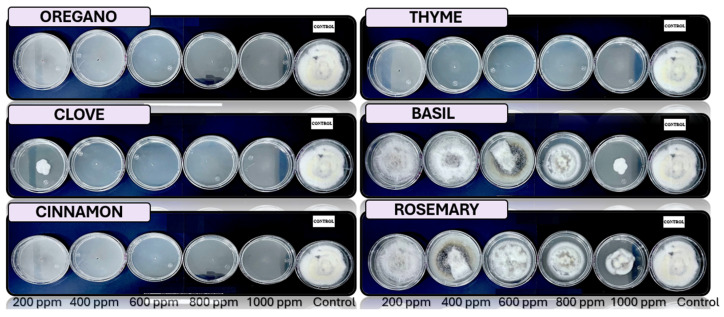
In vitro analysis of *Colletotrichum tengchongense* in PDA medium supplied with basil, cinnamon, clove, oregano, rosemary, and thyme essential oils at various concentrations of 200, 400, 600, 800, and 1000 ppm (*n* = 12).

**Figure 7:**
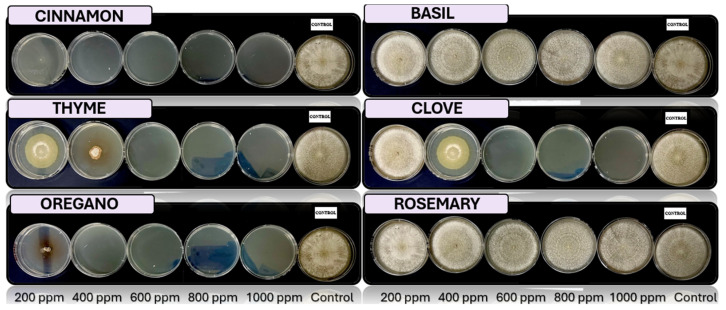
In vitro analysis of *Fusarium verticilloides* in PDA medium supplied with basil, cinnamon, clove, oregano, rosemary, and thyme essential oils at various concentrations of 200, 400, 600, 800, and 1000 ppm (*n* = 12).

**Figure 8:**
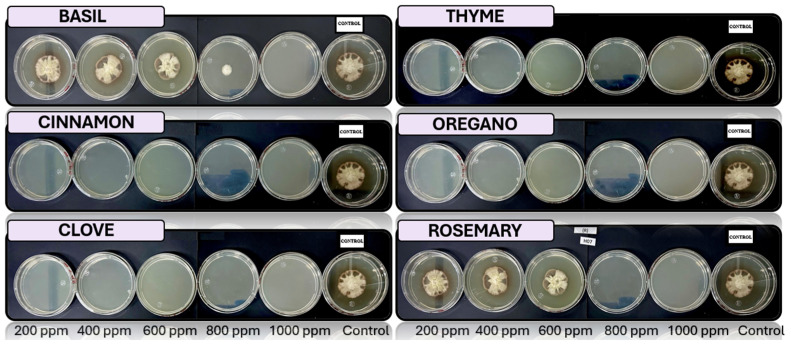
In vitro analysis of *Fusarium napiforme* in PDA medium supplied with basil, cinnamon, clove, oregano, rosemary, and thyme essential oils at various concentrations of 200, 400, 600, 800, and 1000 ppm (*n* = 12).

**Figure 9:**
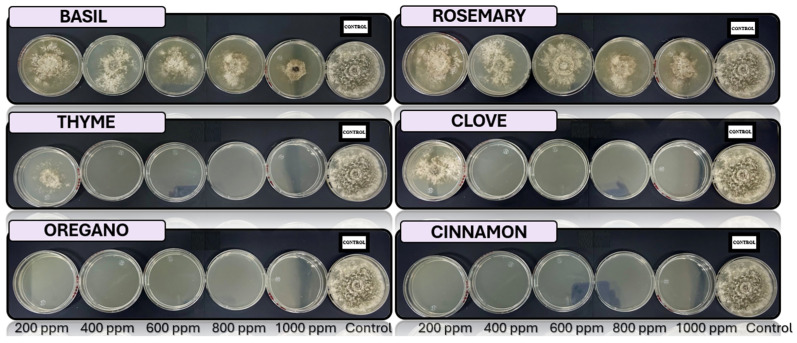
In vitro analysis of *Verticillium dahliae* in PDA medium supplied with basil, cinnamon, clove, oregano, rosemary, and thyme essential oils at various concentrations of 200, 400, 600, 800, and 1000 ppm (*n* = 12).

Figure 5, Figure 6, Figure 7, Figure 8 and Figure 9 illustrate the growth of *Fusarium pseudocircinatum*, *Colletotrichum tengchongense*, *Fusarium napiforme*, *Fusarium verticilloides*, and *Verticillium dahliae*, in PDA medium with EOs concentrations of 200, 400, 600, 800, and 1000 ppm. The EOs evaluated include oregano, basil, cinnamon, rosemary, thyme, and clove, showing their effects on fungal growth at different concentrations.

The antifungal activity of EOs was evaluated in vitro for oregano (*Origanum vulgare*), rosemary (*Salvia rosmarinus*), clove (*Syzygium aromaticum*), thyme (*Thymus vulgaris*), cinnamon (*Cinnamomum verum*), and basil (*Ocimum basilicum*). Table 4 presents the in vitro evaluation of antifungal activity, “+” sign indicates fungal growth, while a “−” sign denotes no fungal growth.

The results obtained with *Fusarium pseudocircinatum* show that oregano oils are highly effective at concentrations above 200 ppm, inhibiting the growth of the fungus in PDA medium. This finding is consistent with previous studies that have demonstrated the antifungal activity of these oils, especially in their ability to control various species of the *Fusarium* genus, known pathogens that cause wilting in bananas [17]. These oils could be useful not only for controlling *Fusarium* in bananas in general, but also as a tool in post-harvest management, where banana peels are particularly vulnerable to infections.

In the case of *Colletotrichum tengchongense*, which causes anthracnose in bananas, it is inhibited con cinnamon, with 400 ppm of clove, oregano, and thyme EOs. This fungus primarily affects the peel, causing black spots and accelerating the fruit’s deterioration, a phenomenon that has been documented in previous studies [45,67]. EOs, particularly cinnamon, have been shown to be effective against various species of *Colletotrichum* in previous studies [68], to managing anthracnose in bananas, and improving post-harvest quality.

*Verticillium dahliae* showed remarkable inhibition by cinnamon, oregano at higher concentrations, and to 200 ppm of thyme and clove EOs. *Verticillium* affects banana plants in the field and may continue to proliferate during storage if not properly controlled [69]. The use of EOs such as oregano, clove, and cinnamon could offer a natural and sustainable alternative to chemical fungicides, with potential benefits for human health and the environment.

The species *Fusarium verticilloides* is sensitive to 400 ppm oregano and cinnamon oils. This fungus is responsible for the rot of the peel, a common problem in the post-harvest stage of bananas. The findings of this study coincide with previous research that has reported the effectiveness of oregano oil in inhibiting several *Fusarium* species [23]. Oregano and cinnamon oils also showed a positive effect against *Fusarium napiforme*, another pathogenic fungus associated with the banana peel. This finding is consistent with previous studies that identified oregano oil as an effective antifungal agent against various *Fusarium* species [70].

## 4. Discussion

### 4.1. Morphological Identification

Regarding the macroscopic analysis in Table 1 and Table 2, the colonies of *Fusarium pseudocircinatum* on PDA medium exhibited a cottony, slightly elevated growth with lobed edges. The surface of the colonies was dry, with a cottony and moderately rough texture, showing colors ranging from beige to gray. The underside of the colony also displayed a cottony texture and similar colors, predominantly white and beige tones. This macroscopic pattern is consistent with previous descriptions of *Fusarium* species [70,71].

In *Colletotrichum tengchongense*, the colonies showed globes to irregular growth, with a cottony texture and colors varying from beige to black. The colonies had a slightly moist or dry surface, with well-defined edges and a somewhat rough appearance. These characteristics are consistent with species of the *Colletotrichum* genus [72]. This growth pattern, with a dense texture and clear color differentiation, helps identify the species within the *Colletotrichum* genus.

For *Fusarium napiforme*, the colonies in the PDA medium exhibited crateriform growth, characterized by a cottony and rough texture on the upper surface. The shape of the colonies was crater-like, with a depressed center surrounded by slightly raised edges. The surface was white to beige, while the underside of the colonies showed darker tones, ranging from brown to dark brown. This crateriform growth pattern, combined with the cottony texture, is distinctive for *Fusarium napiforme* [70]. The elevated and lobed colony appearance, along with the darker coloration of the bottom side, helps in distinguishing this species from other *Fusarium* species [73].

For *Fusarium verticilloides*, the colonies on PDA displayed cottony growth with a rough and elevated texture. The colonies were typically white to beige, with central areas darkening to brown or black as the colony aged. This growth pattern is characteristic of *Fusarium verticilloides* [74]. Finally, for *Verticillium dahliae*, the colonies on PDA exhibited cottony growth with a soft texture, with a circular shape and well-defined edges. The color varied from white on the outer areas to darker tones, such as black, in the center of the colony. This color variation is typical of *Verticillium dahliae* [12,75].

The microscopic analysis provided additional information for the identification of these species. *Fusarium pseudocircinatum* showed hyaline, septate, and thin hyphae, with conidia that were cylindrical to slightly curved, organized in branched conidiophores, typical of this species [70]. In *Colletotrichum tengchongense*, microscopic observations revealed hyaline, septate hyphae, and conidia that were cylindrical to curved, arranged in branched conidiophores, which is a distinguishing feature of the *Colletotrichum* genus [76]. The arrangement of hyphae and conidia is helpful for confirming the species identity, as it is consistently seen in other species of this genus [77].

For *Fusarium verticilloides*, the hyphae were hyaline and septate, while the conidia had a fusiform shape, which is typical for this species [70]. The conidia were organized in branched conidiophores, a feature for identifying *Fusarium* species. In *Fusarium napiforme*, the hyphae were hyaline and septate, and the conidia observed were fusiform or ellipsoidal; the arrangement of conidia along the hyphae and their fusiform [70]. In *Verticillium dahliae*, the microscopic observations revealed hyaline, septate hyphae, and conidia that were cylindrical or oval, arranged in branched structures [75]. The branched conidial structure is a distinctive feature of *Verticillium*.

### 4.2. Molecular Identification Through DNA Sequencing

Molecular sequencing of the ITS fragment provided additional confirmation for the identification of the studied species. *Fusarium pseudocircinatum* showed a 99.54% identity, indicating high concordance with reference sequences in the databases, confirming the molecular identification of the species [74]. This high identity percentage further strengthens the accuracy of macroscopic and microscopic analyses.

For *Colletotrichum tengchongense*, sequencing revealed a 99.66% identity, validating the molecular identification of this species [67]. The high degree of identity with reference sequences indicates that the macroscopic and microscopic results obtained are consistent with the molecular data. *Fusarium verticilloides*, the sequencing analysis of the ITS revealed a 99.01% identity, confirming the correct molecular identification of the species [74].

For *Fusarium napiforme*, the ITS sequencing showed a 98.71% identity, further corroborating the molecular identification of this species; the comparison with reference sequences allowed for the confirmation of its identity [78]. Finally, *Verticillium dahliae* showed a 99.38% identity in the ITS analysis, which also validated the correct molecular identification of the species [69].

The high ITS sequence similarity between fungal isolates and their corresponding GenBank reference strains—exceeding 98.7% in all cases—strongly supports accurate species-level identification. These values are consistent with prior studies that establish a 97–98% identity threshold as reliable for species delimitation using the ITS region [1,2]. The near-complete matches observed for *Colletotrichum tengchongense* (99.66%), *Fusarium pseudocircinatum* (99.54%), and *Fusarium verticillioides* (99.01%) reinforce the taxonomic validity of the isolates and highlight the stability of the ITS marker among fungal pathogens of tropical crops.

In addition, the genetic distance matrix revealed minimal divergence (<0.01) between local isolates and their reference strains, corroborating both the authenticity of the obtained sequences and their phylogenetic positioning. This pattern aligned with the topology of the Neighbor-Joining tree (Figure 3), where four of the five clades displayed bootstrap support values above 93%, indicating robust statistical confidence. The close grouping between H1 and *F. pseudocircinatum*, as well as between H3 and *C. tengchongense*, underscores the effectiveness of ITS sequences as a high-resolution molecular tool for fungal taxonomy.

Altogether, these findings not only validate the species identity of fungal isolates associated with *Musa paradisiaca* but also provide phylogenetic evidence that may be valuable for future studies on fungal diversity, pathogenicity, and biotechnological control in postharvest systems.

### 4.3. Ex Vivo Fungal Activity

This study evaluated the weekly growth of five fungal species inoculated into bananas, observing variability in both the rate of growth and the severity of colonization over time. *Fusarium pseudocircinatum* displayed growth reaching approximately 0.6 cm at six weeks. This suggests that, while not the most aggressive species, it remains a significant pathogen in banana storage contexts. The slow growth observed in the initial weeks, followed by a steady increase, is consistent with its ability to adapt to the environment before proliferating. The relatively high standard deviations indicate some variability in growth between the inoculated samples, which could reflect heterogeneity in storage conditions or intrinsic biological variability of the species [79]. This behavior is also in line with previous studies, which suggest that species of *Fusarium*, such as *Fusarium pseudocircinatum*, can induce decay when environmental conditions are not adequately controlled [70].

*Colletotrichum tengchongense* exhibited the fastest growth among all species studied. By week six, it reached approximately 0.9 cm, standing out for its aggressiveness in colonization. This pattern is characteristic of *Colletotrichum* species, known for their ability to produce rapidly dispersing spores that efficiently colonize plant tissue, especially under high humidity conditions [80,81]. The growth graph shows relatively low standard deviations, suggesting that this pathogen colonized the samples more uniformly. This behavior aligns with previous research that highlights *Colletotrichum* as one of the most aggressive pathogens in tropical fruits like banana, due to its high ability to produce enzymes that degrade plant tissue [65,77].

*Fusarium napiforme* presented intermediate growth, reaching approximately 0.5 cm by the end of six weeks. Its colonization rate was not as rapid as *Colletotrichum tengchongense*, but it remained constant throughout the observation period. The standard deviations suggest that, while their growth was slower compared to the more aggressive species, the inoculated samples showed a relatively uniform response. Describes *Fusarium napiforme* as a less invasive pathogen than other *Fusarium* species, but one that can still cause significant damage under improper storage conditions [78].

*Fusarium verticilloide* exhibited the fastest growth from week two, reaching a colony size of approximately 0.8 cm by week six. Its ability to rapidly adapt to storage conditions is highlighted by its acceleration in the growth rate, making it a high-priority pathogen for banana postharvest management. The growth graph shows a marked acceleration toward the later weeks, with higher standard deviations during the initial stages, which could reflect variability in initial colonization. The growth stabilized, further underscoring its ability to rapidly adapt and occupy the available substrate [74].

*Verticillium dahliae* the least growth among the species, with limited colonization at approximately 0.4 cm by the end of six weeks. This reflects its reduced ability to colonize banana tissue under storage conditions. While *Verticillium dahliae* is a known pathogen in other crops, its slower growth in bananas could indicate that it is less efficient in colonizing tropical fruits compared to other species like *Colletotrichum* or *Fusarium*. The standard deviations suggest that the growth of *Verticillium dahliae* was relatively uniform among the inoculated samples, but its lower aggressiveness could make it a less immediate threat in banana [69].

The study highlights the importance of considering the specific biological characteristics of each fungal species when developing control strategies for banana storage. The more aggressive species, such as *Colletotrichum tengchongense* and *Fusarium verticilloides*, exhibited rapid and sustained growth, emphasizing the need for more stringent control measures to prevent colonization and decay. Conversely, species such as *Fusarium pseudocircinatum* and *Fusarium napiforme*, while less aggressive, remain relevant in terms of their potential for proliferation under inadequate storage conditions.

### 4.4. In Vitro Antifungal Activity with Essential Oils

The antifungal effects observed in this study are primarily attributed to the ability of EOs to disrupt fungal cell membranes and interfere with essential metabolic processes [82]. Compounds such as carvacrol and thymol, present in oregano and thyme EOs, integrate into the lipid bilayer of fungal cell membranes, increasing their permeability. This alteration leads to the loss of ions, metabolites, and essential proteins, ultimately resulting in cell death.

Additionally, cinnamaldehyde and eugenol, found in cinnamon and clove EOs, act by denaturing intracellular proteins, including enzymes essential for cellular metabolism, thereby interfering with fundamental processes such as cellular respiration and ATP synthesis [28]. Some compounds, such as rosmarinic acid in rosemary EOs and eugenol in basil EOs, induce the generation of reactive oxygen species (ROS), causing oxidative damage to DNA, proteins, and cell membrane lipids, which accelerates programmed cell death.

Another significant mechanism is the inhibition of ergosterol biosynthesis, a critical component of the fungal cell membrane. Cinnamaldehyde and carvacrol disrupt this pathway, compromising membrane structure and impairing the transport of essential nutrients, thereby contributing to their antifungal efficacy.

### 4.5. Statistical Analysis

Statistical analyses were conducted to evaluate the effects of fungal species, time (weeks), and treatment conditions on radial mycelial growth. A two-way analysis of variance (ANOVA) was performed to assess the main effects and interaction between species and time. When significant differences were identified (*p* < 0.05), multiple comparisons were carried out using Tukey’s Honestly Significant Difference (HSD) test to identify statistically distinct groups.

Additionally, a one-way ANOVA followed by Tukey’s post hoc test was applied to compare species at the final evaluation point (week 6). Data normality and homogeneity of variances were verified using the Shapiro–Wilk and Levene’s tests, respectively. The significance level was set at α = 0.05 for all analyses.

Pairwise identity matrices and genetic distances were also calculated based on ITS sequences using the Tamura–Nei substitution model in MEGA version 11. Phylogenetic tree topologies were evaluated with 1000 bootstrap replicates, and bootstrap values ≥ 70% were considered indicative of reliable clade support.

## 5. Conclusions

The fungal species identified (*Fusarium pseudocircinatum*, *Colletotrichum tengchongense*, *Fusarium napiforme*, *Fusarium verticilloides*, and *Verticillium dahliae*) exhibit varying levels of aggressiveness. *Colletotrichum tengchongense* and *Fusarium verticilloides* are the most invasive, while *Fusarium pseudocircinatum* and *Fusarium napiforme* grow more slowly but remain relevant under improper storage conditions, and *Verticillium dahliae* showed the most limited growth.

The EOs of oregano (*Origanum vulgare*) and cinnamon (*Cinnamomum verum*) effectively inhibited fungal growth at a concentration of 600 ppm.

The EOs of rosemary (*Salvia rosmarinus*), basil (*Ocimum basilicum*), and clove (*Syzygium aromaticum*) did not inhibit the fungal growth of *Fusarium* spp., *Colletotrichum* spp., and *Verticillium* spp. at the concentrations evaluated.

The results of this study suggest that EOs, such as oregano, cinnamon, and clove, are effective natural alternatives for controlling postharvest banana diseases. These oils showed significant antifungal potential and can be incorporated into sustainable agricultural management strategies.

A limitation of this study is the focus on a limited range of EOs and fungal species. Future research should explore additional EOs and their efficacy against a broader spectrum of fungal species, as well as assess their long-term effectiveness in real-world storage conditions.

## Figures and Tables

**Table 1:** Experimental conditions for the evaluation of essential oils against banana fungi.

Parameter	Details
Fungal species	*Fusarium pseudocircinatum*, *Colletotrichum tengchongense*, *Fusarium napiforme*, *Fusarium verticilloides*, *Verticillium dahliae*
Source of fungal isolates	Infected banana peel samples from Ecuadorian *Musa paradisiaca*
Inoculum preparation	10^6^ conidia/mL for each fungal species
Medium used	Potato Dextrose Agar (PDA) supplemented with 0.05% chloramphenicol to prevent bacterial growth
Essential oils tested	Oregano (*Origanum vulgare*), Rosemary (*Salvia rosmarinus*), Clove (*Syzygium aromaticum)*, Thyme (*Thymus vulgaris*), Cinnamon (*Cinnamomum verum*), Basil (*Ocimum basilicum*)
Essential oil concentrations	200, 400, 600, 800, 1000 ppm
Inoculation method	Wound method: 100 µL of adjusted fungal inoculum applied to the banana fruit
Incubation conditions	At 25 ± 2 °C and 75 ± 5% relative humidity, 24 h monitoring intervals for in vitro assays; at approximately 13 ± 1 °C and 92 ± 3% relative humidity for ex vivo assays
Control treatment	PDA medium without essential oils (negative control)
Duration of observation	6 weeks for ex vivo assays, 20 wounds per fungal species, and 24 h for in vitro assays

**Table 2:** Sequencing results for fungi isolated from the samples of *Musa paradisiaca.*

Organism	Fragment	NCBI	% Identity
*Fusarium pseudocircinatum*	ITS	MG838060.1	99.54%
*Fusarium napiforme*	ITS	ON204349.1	98.71%
*Colletotrichum tengchongense*	ITS	OL842169.1	99.66%
*Fusarium verticilloides*	ITS	PQ416097.1	99.01%
*Verticillium dahliae*	ITS	NR 126124.1	99.38%

**Table 3:** Pairwise genetic distance matrix of ITS sequences among fungal isolates and GenBank reference strains.

	MG838060.1	H1	ON204349.1	H2	OL842169.1	H3	PQ416097.1	H4	NR_126124.1	H5
MG838060.1	ID	0.002	2.439	2.704	2.949	2.947	2.492	2.511	2.293	2.294
H1	0.005	ID	2.439	2.704	2.949	2.947	2.492	2.511	2.293	2.294
ON204349.1	2.752	2.752	ID	2.251	2.401	2.366	2.298	2.308	2.345	2.333
H2	5.106	5.106	2.524	ID	2.695	2.676	2.609	2.528	2.306	2.295
OL842169.1	5.62	5.62	3.998	4.639	ID	0.002	2.124	1.943	2.468	2.575
H3	5.656	5.656	4.075	4.671	0.003	ID	2.124	1.943	2.468	2.575
PQ416097.1	4.511	4.511	3.786	4.445	2.397	2.397	ID	0.005	2.3	2.302
H4	4.393	4.393	3.778	3.829	2.401	2.401	0.01	ID	2.3	2.302
NR_126124.1	3.52	3.52	3.871	4.226	5.121	5.121	3.683	3.683	ID	0.004
H5	3.499	3.499	3.902	4.358	5.193	5.193	3.66	3.66	0.006	ID

**Table 4:** Evaluation of antifungal activity in vitro of *Fusarium pseudocircinatum*, *Fusarium verticilloides*, *Colletotrichum tengchongense*, *Fusarium napiforme*, and *Verticillium dahliae* using oregano, rosemary, clove, thyme, cinnamon, and basil EOs.

*Essential Oil*	Fungus	Concentration [ppm]				
		200	400	600	800	1000
Cinnamon	*Fusarium pseudocircinatum*	+	+	−	−	−
	*Colletotrichum tengchongense*	−	−	−	−	−
	*Fusarium verticilloides*	+	−	−	−	−
	*Fusarium napiforme*	−	−	−	−	−
	*Verticillium dahliae*	−	−	−	−	−
Clove	*Fusarium pseudocircinatum*	+	+	+	+	+
	*Colletotrichum tengchongense*	+	+	−	−	−
	*Fusarium verticilloides*	+	+	−	−	−
	*Fusarium napiforme*	−	−	−	−	−
	*Verticillium dahliae*	+	−	−	−	−
Basil	*Fusarium pseudocircinatum*	+	+	+	+	+
	*Colletotrichum tengchongense*	+	+	+	+	+
	*Fusarium verticilloides*	+	+	+	+	+
	*Fusarium napiforme*	+	+	+	+	−
	*Verticillium dahliae*	+	+	+	+	+
Oregano	*Fusarium pseudocircinatum*	+	−	−	−	−
	*Colletotrichum tengchongense*	+	+	−	−	−
	*Fusarium verticilloides*	+	−	−	−	−
	*Fusarium napiforme*	−	−	−	−	−
	*Verticillium dahliae*	−	−	−	−	−
Rosemary	*Fusarium pseudocircinatum*	+	+	+	+	+
	*Colletotrichum tengchongense*	+	+	+	+	+
	*Fusarium verticilloides*	+	+	+	+	+
	*Fusarium napiforme*	+	+	+	−	−
	*Verticillium dahliae*	+	+	+	+	+
Thyme	*Fusarium pseudocircinatum*	+	+	+	+	−
	*Colletotrichum tengchongense*	+	+	−	−	−
	*Fusarium verticilloides*	+	+	−	−	−
	*Fusarium napiforme*	−	−	−	−	−
	*Verticillium dahlia*	+	−	−	−	−

## Data Availability

The original contributions presented in this study are included in the article. Further inquiries can be directed to the corresponding author.

## References

[1] Tang Q., Liu D., Zhang J. (2019). Post-harvest control of fungal diseases in bananas using essential oils. J. Postharvest Biol..

[2] Mata Anchundia D., Suatunce Cunuhay P., Poveda Morán R. (2021). Análisis económico del banano orgánico y convencional en la provincia Los Ríos, Ecuador. Avances.

[3] Borges C.V., Amorim E.P., Leonel M., Gomez Gomez H.A., Santos T.P.R.d., Ledo C.A.d.S., Belin M.A.F., Almeida S.L.d., Minatel I.O., Lima G.P.P. (2018). Post-harvest physicochemical profile and bioactive compounds of 19 bananas and plantains genotypes. Bragantia.

[4] Vallejo-Rojas V., Rivera-Ferre M.G., Ravera F. (2022). The agri-food system (re)configuration: The case study of an agroecological network in the Ecuadorian Andes. Agric. Hum. Values.

[5] Alvindia D.G., Natsuaki K.T. (2008). Biocontrol of crown rot-causing Colletotrichum musae by *Burkholderia* sp.. Crop Prot..

[6] Ruiz Medina M.D., Ruales J. (2024). Postharvest Alternatives in Banana Cultivation. Agronomy.

[7] Aguilar-Anccota R., Arévalo-Quinde C.G., Morales-Pizarro A., Galecio-Julca M. (2021). Hongos asociados a la necrosis de haces vasculares en el cultivo de banano orgánico: Síntomas, aislamiento e identificación, y alternativas de manejo integrado. Sci. Agropecu..

[8] Capa Benítez L.B., Alaña Castillo T.P., Benítez Narváez R.M. (2016). Importancia de la producción de banano orgánico. Caso: Provincia de El Oro, Ecuador. Rev. Univ. Y Soc..

[9] Agronomía C. (2005). Tecnología Poscosecha. Agron. Costarric..

[10] Mari M., Torres R., Vanneste J.L. (2014). Biological control of postharvest diseases: Opportunities and challenges. Front. Microbiol..

[11] Villa-Martínez A., Pérez-Leal R., Morales-Morales H.A., Basurto-Sotelo M., Soto-Parra J.M., Martínez-Escudero E. (2015). Situación actual en el control de Fusarium spp. y evaluación de la actividad antifúngica de extractos vegetales. Acta Agronómica.

[12] Goicoechea N. (2009). To what extent are soil amendments useful to control Verticillium wilt?. Pest Manag. Sci..

[13] Pabon Montoya B., Córdova Chávez M., Alban Alcivar J., Jaramillo Robles A. (2024). Efectos antifúngicos de extractos botánicos sobre el crecimiento micelial de *Colletotrichum* sp. a nivel in vitro, causante de antracnosis en la fruta de aguacate. 593 Digit. Publ. CEIT.

[14] Alzate D., Mier G., Afanador L., Durango D., García C. (2009). Evaluación de la fitotoxicidad y la actividad antifúngica contra Colletotrichum acutatum de los aceites esenciales de tomillo (*Thymus vulgaris*), limoncillo (*Cymbopogon citratus*), y sus componentes mayoritarios. Vitae.

[15] Bandoni A.L., Retta D., Lira P.M.D.L., van Baren C.M. (2009). ¿Son realmente útiles los aceites esenciales?. Boletín Latinoam. Y Caribe Plantas Med. Y Aromáticas.

[16] Oliva M.D.L.M., Lorello I.M., Baglio C., Posadaz A., Carezzano M.E., Paletti Rovey M.F., Huallpa C.L., Juliani H.R. (2023). Chemical composition, antioxidant, and antimicrobial activities of rosemary (*Salvia rosmarinus* Spenn.) essential oils from Argentina. J. Med. Act. Plants.

[17] Pilozo G., Villavicencio-Vásquez M., Chóez-Guaranda I., Murillo D.V., Pasaguay C.D., Reyes C.T., Maldonado-Estupinan M., Ruiz-Barzola O., Leon-Tamariz F., Manzano P. (2024). Chemical, antioxidant, and antifungal analysis of oregano and thyme essential oils from Ecuador: Effect of thyme against *Lasiodiplodia theobromae* and its application in banana rot. Heliyon.

[18] Flores-Villa E., Sáenz-Galindo A., Castañeda-Facio A.O., Narro-Céspedes R.I. (2020). Romero (*Rosmarinus officinalis* L.): Su origen, importancia y generalidades de sus metabolitos secundarios. TIP Rev. Espec. En Cienc. Químico-Biológicas.

[19] Farias A.P.P., Monteiro Odos S., da Silva J.K.R., Figueiredo P.L.B., Rodrigues A.A.C., Monteiro I.N., Maia J.G.S. (2020). Chemical composition and biological activities of two chemotype-oils from *Cinnamomum verum* J. Presl growing in North Brazil. J. Food Sci. Technol..

[20] Pinto E., Silva C., Costa L. (2018). Eugenol as an antifungal agent: Mechanisms and applications. J. Appl. Microbiol..

[21] Ruiz Medina M.D., Quimbita Yupangui Y., Ruales J. (2025). Effect of a Protein–Polysaccharide Coating on the Physicochemical Properties of Banana (*Musa paradisiaca*) During Storage. Coatings.

[22] Abadias M., Teixidó N., Usall J., Viñas I. (2008). Evaluation of alternative strategies to control postharvest blue mould of apple caused by Penicillium expansum. Int. J. Food Microbiol..

[23] Burt S. (2004). Essential oils: Their antibacterial properties and potential applications in foods—A review. Int. J. Food Microbiol..

[24] Mohamed S., Saban K. (2019). Review of essential oils as anti-fungal agents for plant fungal diseases. Ziraat Fakültesi Dergisi..

[25] Zhang W., Li B., Lv Y., Wei S., Zhang S., Hu Y. (2023). Synergistic effects of combined cinnamaldehyde and nonanal vapors against *Aspergillus flavus*. Int. J. Food Microbiol..

[26] Haro-González J.N., Castillo-Herrera G.A., Martínez-Velázquez M., Espinosa-Andrews H. (2021). Clove Essential Oil (*Syzygium aromaticum* L. Myrtaceae): Extraction, Chemical Composition, Food Applications, and Essential Bioactivity for Human Health. Molecules.

[27] Kintzios S.E., Peter K.V. (2012). Oregano. Handbook of Herbs and Spices.

[28] Nazzarro F., Fratianni F., Coppola R., De Feo V. (2017). Essential Oils and Antifungal Activity. Pharmaceuticals.

[29] Arcila-Lozano C.C., Loarca-Piña G., Lecona-Uribe S., González de Mejía E. (2004). El orégano: Propiedades, composición y actividad biológica de sus componentes. Arch. Latinoam. Nutr..

[30] Prashar A., Locke I.C., Evans C.S. (2006). Cytotoxicity of clove (*Syzygium aromaticum*) oil and its major components to human skin cells. Cell Prolif..

[31] Selles S.M.A., Kouidri M., Belhamiti B.T., Ait Amrane A. (2020). Chemical composition, in-vitro antibacterial and antioxidant activities of Syzygium aromaticum essential oil. Food Meas..

[32] Ruiz Medina M., Ávila J., Ruales J. (2016). Diseño de un recubrimiento comestible bioactivo para aplicarlo en la frutilla (*Fragaria vesca*) como proceso de postcosecha. Rev. Iberoam. Tecnol. Postcosecha.

[33] Aquino-Martínez J.G., Vázquez-García L.M., Reyes-Reyes B.G. (2008). Biocontrol in vitro e in vivo de *Fusarium oxysporum* Schlecht. f. sp. dianthi (Prill. y Delacr.) Snyder y Hans. con Hongos Antagonistas Nativos de la Zona Florícola de Villa Guerrero, Estado de México. Rev. Mex. Fitopatol..

[34] Li W., Li G., Zhang H. (2021). Postharvest disease of banana caused by *Fusarium musae*: A public health concern. J. Appl. Microbiol..

[35] Barrera Necha L.L., García Barrera L.J. (2008). Actividad antifúngica de aceites esenciales y sus compuestos sobre el crecimiento de Fusarium sp. aislado de papaya (*Carica papaya*). Rev. Científica UDO Agrícola.

[36] Ultee A., Bennik M.H.J., Moezelaar R. (2002). The phenolic hydroxyl group of carvacrol is essential for action against the food-borne pathogen *Bacillus cereus*. Appl. Environ. Microbiol..

[37] Pina-Vaz C., Gonçalves Rodrigues A., Pinto E., Costa-de-Oliveira S., Tavares C., Salgueiro L., Cavaleiro C., Gonçalves M.J., Martinez-de-Oliveira J. (2004). Antifungal activity of *Thymus* oils and their major compounds. J. Eur. Acad. Dermatol. Venereol..

[38] Samarakoon K.W., Thong P.H., Jeewanthi R.K.C. (2020). Evaluation of antifungal activity of cassia and holy basil essential oils against postharvest banana pathogens. Chem. Pap..

[39] Shahrajabian M.H., Sun W., Cheng Q. (2020). Chemical components and pharmacological benefits of Basil (*Ocimum basilicum*): A review. Int. J. Food Prop..

[40] Salazar E., Hernández R., Tapia A., Gómez-Alpízar L. (2012). Identificación molecular del hongo *Colletotrichum* spp., aislado de banano (*Musa* spp.) de la altura en la zona de Turrialba y determinación de su sensibilidad a fungicidas poscosecha. Agron. Costarric..

[41] Tortora G.J., Funke B.R., Case C.L. (2007). Introducción a la Microbiología.

[42] Agu K., Awah N., Nnadozie A. (2016). Isolation, identification, and pathogenicity of fungi associated with cocoyam (*Colocasia esculenta*) spoilage. Food Sci. Technol..

[43] Suárez L., Rangel A. (2013). Aislamiento de microorganismos para control biológico de Moniliophthora roreri. Acta Agronómica.

[44] Orwa P., Mugambi G., Wekesa V., Mwirichia R. (2020). Isolation of haloalkaliphilic fungi from Lake Magadi in Kenya. Heliyon.

[45] Vilaplana R., Pazmiño L., Valencia-Chamorro S. (2018). Control of anthracnose, caused by *Colletotrichum musae*, on postharvest organic banana by thyme oil. Postharvest Biol. Technol..

[46] Morales R., Henríquez G. (2021). Aislamiento e identificación del moho causante de antracnosis en musa paradisiaca l. (plátano) en cooperativa san carlos, el salvador y aislamiento de mohos y levaduras con capacidad antagonista. Crea Cienc. Rev. Científica.

[47] Funnell-Harris D., Prom L. (2013). Isolation and characterization of grain mold fungi Cochliobolus and Alternaria spp. from sorghum using semiselective media and DNA sequence analyses. Can. J. Microbiol..

[48] Vargas-Fernández J.P., Wang-Wong A., Muñoz-Fonseca M. (2022). Microorganismos asociados a la enfermedad conocida como pudrición suave del fruto de banano (*Musa* sp.) y alternativas de control microbiológicas y químicas a nivel in vitro *. Agron. Costarric..

[49] Ruiz Medina M.D., Ruales J. (2025). Aceites esenciales como alternativa antifúngica para el control de diversas especies de hongos aislados de *Musa paradisiaca*: Parte I. Preprints.

[50] Johanna S.V., Natalia C.G., Ximena Carolina P.M. (2014). Manual de Microbiología General: Principios Básicos de Laboratorio.

[51] Espinel-Ingroff A., Montero D., Martin-Mazuelos E. (2004). Long-term preservation of fungal isolates in commercially prepared cryogenic microbank vials. J. Clin. Microbiol..

[52] Siller-Ruiz M., Hernández-Egido S., Sánchez-Juanes F., González-Buitrago J.M., Muñoz-Bellido J.L. (2017). Métodos rápidos de identificación de bacterias y hongos. Enfermedades Infecc. Microbiol. Clínica.

[53] Acurio Vásconez R.D., España Imbaquingo C.K., Acurio Vásconez R.D., España Imbaquingo C.K. (2017). Aislamiento, caracterización y evaluación de *Trichoderma* spp. como promotor de crecimiento vegetal en pasturas de Raygrass (*Lolium perenne*) y trébol blanco (*Trifolium repens*). LA GRANJA Rev. Cienc. De La Vida.

[54] Carpio-Coba C.F., Noboa-Silva V.F., Salazar-Castañeda E.P., Lema-Saigua E.R. (2021). Caracterización macroscópica y microscópica de cuatro especies forestales de la amazonia del sur de Ecuador. Polo Del Conoc..

[55] Bellemain E., Carlsen T., Brochmann C., Coissac E., Taberlet P., Kauserud H. (2010). ITS as an environmental DNA barcode for fungi: An in silico approach reveals potential PCR biases. BMC Microbiol..

[56] Cervantes C., Tarqui M., Huiza P., Quispe A. Determinación de Secuencias de ADN en Bioedit. ResearchGate n.d. https://www.researchgate.net/publication/370895529_Determinacion_de_secuencias_de_ADN_en_Bioedit.

[57] Tamura K., Stecher G., Kumar S. (2021). Molecular Evolutionary Genetics Analysis version 11. Mol. Biol. Evol..

[58] Zhang M., Zhao J., Dai X., Li X. (2023). Extracción y análisis de composiciones químicas de productos naturales y plantas. Separations.

[59] National Institute of Standards and Technology (2011). Essential Oils: The Effects of Processing and Standards for Quality Control.

[60] Cavanagh H.M., Wilkinson J.M. (2002). Biological activities of lavender essential oil. Phytother. Res..

[61] Baser K.H.C., Buchbauer G. (2015). Handbook of Essential Oils: Science, Technology, and Applications.

[62] Bakkali F., Averbeck S., Averbeck D., Idaomar M. (2008). Biological effects of essential oils—A review. Food Chem. Toxicol..

[63] Letseka T.E., Sepheka N.J., Dubery I.A., George M.J. (2022). Bioprospecting of Essential Oil-Bearing Plants: Rapid Screening of Volatile Organic Compounds Using Headspace Bubble-in-Drop Single-Drop Microextraction for Gas Chromatography Analysis. Plants.

[64] Chacón-Cascante A., Crespi J.M. (2006). Historical overview of the European Union banana import policy. Agron. Costarric..

[65] Aguilar-Anccota R., Apaza-Apaza S., Maldonado E., Calle-Cheje Y., Rafael-Rutte R., Montalvo K. (2024). Control in vitro e in vivo de Thielavipsis paradoxa y Collettrichum musae cn biofungicidas en frutos de banano orgánico. Manglar.

[66] Magri A., Curci M., Battaglia V., Fiorentino A., Petriccione M. (2023). Essential Oils in Postharvest Treatment against Microbial Spoilage of the *Rosaceae* Family Fruits. AppliedChem.

[67] Hassan O., Jong Y., Taehyun C., Jun S. (2018). Molecular and morphological characterization of Colletotrichum species in the Colletotrichum gleosporoides. Plant Dis..

[68] Ranasinghe L., Jayawardena B., Abeywickrama K. (2002). Fungicidal activity of essential oils of *Cinnamomum zeylanicum* (L.) and *Syzygium aromaticum* (L.) Merr et LM Perry Against Crown Rot Anthracnose pathogens Isol. banana. Lett. Appl. Microbiol..

[69] Jabnoun-Khiareddine H., Mahjoub M., Daami-Remadi M. (2010). Morphological Variability Within and Among Verticillium Species Collected in Tunisia. Tunis. J. Plant Prot..

[70] Nuangmek W., Kumla J., Khuna S., Lumyong S., Suwannarach N. (2023). Identificación y caracterización de especies *de Fusarium* causantes de la pudrición de la sandía en el norte de Tailandia. Plants.

[71] Leslie J.F., Summerell B.A. (2006). The Fusarium Laboratory Manual.

[72] Yang R., Li Y., Zhao H., Sun X., Chen W., Li P., Li X., Wu C., Ma M., Gong G. (2024). Identificación y caracterización de especies *de Colletotrichum* asociadas con el maíz en Sichuan, China. J. Fungi.

[73] Carrasco A., Sanfuentes E., Duran A., Valenzuela S. (2016). Cancro resinoso del pino: ¿una amenaza potencial para las plantaciones de Pinus radiata en Chile?. Gayana Botánica.

[74] Sherif M., El-Debaiky S.A., El-Samawaty A.R.M., Baka Z.A.M., Elsharkawy M.M., El-Bebany A.F. (2023). The Role of Mycotoxins in Interactions between *Fusarium graminearum* and *F. verticillioides* Growing in Saprophytic Cultures and Co-Infecting Maize Plants. Toxins.

[75] Liu L., Zhang Y.-D., Zhang D.-D., Zhang Y.-Y., Wang D., Song J., Zhang J., Li R., Kong Z.-Q., Klosterman S.J. (2021). Características biológicas de las cepas MAT1-1 y *MAT1-2 de Verticillium dahliae*. Int. J. Mol. Sci..

[76] Hariharan G., Prasannath K. (2021). Recent Advances in Molecular Diagnostics of Fungal Plant Pathogens: A Mini Review. Front. Cell. Infect. Microbiol..

[77] Rojo-Báez I., Álvarez-Rodríguez B., García-Estrada R., León-Félix J., Sañudo Barajas J.A., Allende R. (2017). Situación actual de *Colletotrichums* spp. en México: Taxonomía, caracterización, patogénesis y control. Rev. Mex. De Fitopatol. Mex. J. Phytopathol..

[78] Ekwomadu T.I., Mwanza M. (2023). Hongos patógenos *de Fusarium*, identificación, efectos adversos, manejo de enfermedades y seguridad alimentaria mundial: Una revisión de las últimas investigaciones. Agricultura.

[79] Perrier X. (2009). Combinando enfoques biológicos para esclarecer la evolución de los plátanos comestibles. Ethnobot. Res. Appl..

[80] Sharma R.R., Singh D., Singh R. (2009). Biological control of postharvest diseases of fruits and vegetables by microbial antagonists: A review. Biol. Control.

[81] Ramudingana P., Makhado N., Kamutando C.N., Thantsha M.S., Mamphogoro T.P. (2025). Agentes de biocontrol fúngico en el manejo de pérdidas poscosecha de productos frescos: Una revisión exhaustiva. J. Fungi.

[82] RuizMedina M., Ruales J. (2025). Aceites esenciales como alternativa antifúngica para el control de diversas especies de hongos aislados de *Musa paradisiaca*: Parte II. Preprints.

